# Survey Showed That Limiting Daily Screen Time Could Help to Avoid Mental Health Issues in Children Aged 8–17Years

**DOI:** 10.1111/apa.70524

**Published:** 2026-03-31

**Authors:** Anders Carlander, Sophie Cassel, Malin J‐son Höök, Oskar Lundgren, Ann‐Sophie Lindqvist Bagge, Marie Löf

**Affiliations:** ^1^ SOM Institute University of Gothenburg Gothenburg Sweden; ^2^ Wallenberg Centre for Molecular and Translational Medicine University of Gothenburg Gothenburg Sweden; ^3^ Generation Pep Stockholm Stockholm Sweden; ^4^ Department of Biomedical and Clinical Sciences Linköping University Linköping Sweden; ^5^ H.K.H. Crown Princess Victoria Children's Hospital Linköping Sweden; ^6^ Department of Psychology University of Gothenburg Sweden; ^7^ Department of Biosciences and Nutrition Karolinska Institutet Stockholm Sweden

**Keywords:** anxiety, depression, mental health, population‐based study, screen time

## Abstract

**Aim:**

This study tested the associations between screen time and anxiety and depression, while accounting for physical activity, sleep and socioeconomic background.

**Methods:**

We analysed repeated cross‐sectional survey data from the Swedish population‐based Generation Pep Study, which was collected in 2021**–**2022 and comprised children aged 8**–**17 years. Screen time and symptoms of anxiety and depression were measured using the short 25‐item child‐reported Revised Children's Anxiety and Depression Scale, along with physical activity, sleep, sex and age. The parents provided some of the basic details and helped younger children under 12 complete the questionnaire, as required.

**Results:**

The 4002 children (50.9% boys) had a mean age of 12.2 ± 2.7 years. The scores for anxiety and depression nearly doubled when the daily screen time increased from approximately two hours to seven hours or more. However, statistically significant marginal effects were only observed when screen time exceeded two hours per day. The results remained significant after adjusting for the plausible displacement effect of physical activity, sleep and socioeconomic status.

**Conclusion:**

Screen time that exceeded about two hours a day was associated with higher levels of anxiety and depression among Swedish children who took part in this population‐based study.

## Summary

- This population–based Swedish study tested the associations between screen time and anxiety and depression in 4002 children from 8‐17 years of age.
- Mental health scores nearly doubled when daily screen time increased from approximately two hours to seven hours or more, but statistically significant marginal effects were only observed when it exceeded two hours.
- The results remained significant after adjusting for the effects of physical activity, sleep and socioeconomic status.

**Abbreviations**
DSM‐V: Diagnostic and Statistical Manual of Mental Disorders, Fifth Edition
RCADS‐25‐C: Revised Children's Anxiety and Depression Scale‐25—Child Report

## 1 Introduction

An umbrella review of 102 meta‐analyses, published in 2024, showed that screen use among children and adolescents was a double‐edged sword of both potential harm and benefits. However, the general finding was that screen use was negatively associated with health outcomes [1].

The Goldilocks hypothesis proposes that just the right amount of screen time may be beneficial for children, but very high and very low levels may be detrimental to their mental health [2]. A number of findings have been reported that are consistent with this hypothesis. Screen time has been reported to be negatively associated with mental health, cognitive function [3] and mental well‐being [4]. It has also been positively associated with aggression, attention deficit hyperactivity disorder, anxiety and depression [5, 6]. However, a review showed that longitudinal associations between screen time and mental health were small to negligible [7].

The World Health Organization defines sedentary behaviour as low energy expenditure, while sitting or lying awake, at school, at home or in community settings and during transport [8]. Reviews have shown that higher levels of sedentary behaviour while using screens were associated with increased mental health problems among children and adolescents [9, 10]. In contrast, several systematic reviews and meta‐analyses demonstrated that physical activity was related to better self‐rated health among children and adolescents aged 3–18 years [11] and improved mental health for children [12, 13]. The ages were not detailed in the last two studies.

Sleep is essential for mental health. A meta‐analysis of randomised controlled trials, which focused on interventions that aimed to improve sleep, demonstrated that enhancing sleep quality contributed to better mental health [14]. According to the Diagnostic and Statistical Manual of Mental Disorders, Fifth Edition (DSM‐V) [15], insomnia can be a symptom of a number of mental disorders or a contributory factor [16].

One explanation for the adverse associations with excessive screen time is the displacement hypothesis, which states that screen time competes with time spent on physical activity, sleep or social interactions [17]. Interestingly, only a few of the studies, systematic reviews and meta‐analyses already mentioned [1, 4, 5, 6, 9, 18] advanced sleep or physical activity as partial explanations for negative relationships between screen time and mental health. However, it is important to acknowledge that screen time may also be beneficial [1]. The Goldilocks hypothesis can be seen as a complementary theory to the displacement hypothesis, by suggesting that there is an optimal amount of positive screen time that can be integrated into a healthy lifestyle without adverse effects [2].

The first aim of this study was to test the displacement hypothesis, by investigating the unique contribution that screen time had on anxiety and depression in children aged 8–17 years. We did this by controlling for physical activity, sleep, sex, age, parents' educational level and household income. Second, we explored the Goldilocks hypothesis, by analysing how different levels of screen time may be associated with anxiety and depression. Our aim was to try and quantify the optimum screen time that correlated with the lowest point of anxiety and depression.

## 2 Methods

### 2.1 Study Design and Participants

This study was based on the cross‐sectional Swedish Generation Pep Study, an online survey on the health of children and adolescents that has been carried out by our team every year since 2018 [19]. A random probability sample of 29 000 children and adolescents aged 4–17 years was drawn from the Swedish population registry in August 2021.

Printed postcards were mailed to the total sample at the start of September 2021, which included information about the online survey and login details. The data were collected between 16 September 2021 and 18 January 2022, and one postal reminder and four text reminders were sent. Consent was received from 7045 individuals (24%), and these comprised the parents of children aged 4–14 and adolescents aged 15–17.

This study focused on the 4002 questionnaires that were completed for children aged 8–17 years. Incomplete questionnaires were excluded from the analysis and we did not include children up to seven years of age because they filled out a different instrument with a poorer model fit.

Although this was a child self‐report survey, children under 12 years of age were encouraged to ask a parent if they needed help to answer the questions. The first part of the questionnaire was aimed at the parents and they filled in details such as their educational attainment and household income.

### 2.2 Measures

The participant's sex and age were validated during the sampling procedure, using the Swedish population registry. The parents' level of educational attainment ranged from elementary for the basic mandatory education in Sweden, which goes up to 15 years of age to a university degree lasting four years or more. Household income ranged from less than 10 000 Swedish Krona to 100 000 or more per month.

We used the short 25‐item child‐report version of the Revised Children's Anxiety and Depression Scale (RCADS‐25‐C), which comprises 15 questions on symptoms of anxiety and 10 on depression [20]. The tool assesses symptoms based on the DSM‐V [15] and has demonstrated good psychometric properties across populations [21]. Carlander et al. [22] validated the Swedish version of the RCADS‐25‐C and confirmed its psychometric reliability. The RCADS‐25‐C includes a series of statements that the subject is asked to answer on a four‐point scale, ranging from zero for never to three for always. These include whether they worry what people think of them, on the anxiety subscale, and whether they feel sad or empty on the depression subscale.

We measured self‐reported screen time the previous day with a single‐item question. This asked how much time they spent in front of a screen outside school lessons and clarified that this covered mobile phones, televisions, computers and tablet screens. The responses used a six‐point scale that ranged from one for not at all to six for seven hours or more.

Self‐reported physical activity was assessed with a single question about how long they were physically active the previous day. The responses used a seven‐point rating that ranged from one for not at all to seven for 2–3 h. This survey question was subsequently assessed by inviting children all from the 2022 Generation Pep Study to take part in an extended study using a three‐axis accelerometer to assess physical activity [23]. We included the first 700 to reply, as that was how many units we had. Self‐reported physical activity was moderately correlated with device‐based physical activity in 434 subjects, as predicted (Spearman's rho = 0.24, *p* < 0.001). This result was expected, as it was consistent with previous validation studies on physical activity in children and adolescents.

Hours of sleep were measured using separate questions about what time the participants usually went to bed and when they woke up during the week and at weekends or holidays. These used pre‐defined time stamps in 30‐min intervals. We used a grand mean average of total sleep hours by combining sleep hours on weekdays and sleep hours on weekends and holidays. This aligned with how the DSM‐V typically defines the duration of sleep problems as a diagnostic symptom or as a risk factor by using weeks and months rather than days [15].

### 2.3 Data Analytic Plan and Initial Results

The children's sex and age are presented as descriptive statistics and the parents' educational attainment as percentages. The descriptive statistics of the main variables of interest are presented as means, standard deviations and internal consistency, using McDonald's omega. There are anxiety and depression using the RCADS‐25‐C, screen time, physical activity and sleep. Pearson correlation coefficients are presented for anxiety, depression and sleep. Spearman‐Rank coefficients are presented for correlations involving screen time and physical activity, due to their ordinal nature.

**FIGURE 1:**
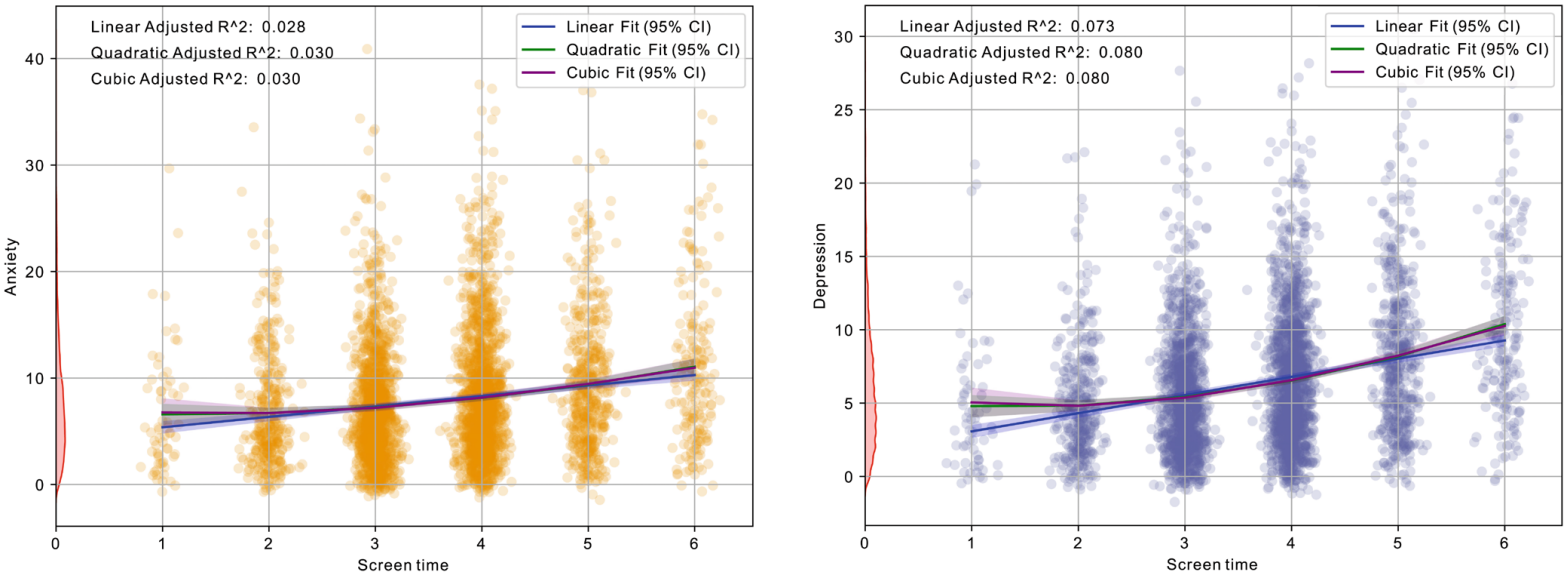
Kernel density estimation plot and scatter plot of screen time and RCADS‐25‐C anxiety and depression: Linear, quadratic and cubic fitted lines jointly with 95% confidence intervals and explained variance (adjusted R^2^).

The initial diagnostic process involved plotting the dispersion of screen time over the anxiety and depression scores. We treated the six‐point ordinal scale of screen time as a continuous scale, so that we could predict anxiety and depression in linear, quadratic and third‐degree polynomial cubic models (Figure 1). These produced similar results when it came to explaining any variance. The Breusch–Pagan/Cook–Weisberg test for heteroskedasticity indicated that the variances were not homogenous for anxiety (chi‐square 1 = 51.99, *p* < 0.001) or depression (chi‐squared 1 = 97.72, *p* < 0.001). Robust standard errors were used for each regression analysis. We investigated the associations between screen time and anxiety and depression using hierarchical linear regression models, including control variables. These were the children's physical activity, sleep, sex and age and the parents' educational attainment and household income. We modelled screen time as an ordinal variable by using marginal effects as our main estimation method.

Analyses were conducted using Stata version 18 (StataCorp LLC, Texas, USA) and Python version 3.11.5 (Python Software Foundation, Delaware, USA), with the Matplotlib version 3.7.2, Seaborn version 0.12.2 and Statsmodels version 0.14.0 extensions.

### 2.4 Ethics

Informed, parental consent was obtained for all the children who were aged 8–14 and from the participants themselves if they were aged 15–17. The 2021 Generation Pep Study was approved by the Swedish Ethical Review Authority (number 2021–03931).

## 3 Results

The study comprised 4002 children and adolescents (51% boys) from 8 to17 years of age and their mean age was 12.2 ± 2.74 years. The results showed that 65% of the parents had a relatively high level of education, as they had attended university for three or more years, which equated to a bachelor's degree. This is regarded as a significant level of educational attainment and is commonly used when comparing to population numbers from Statistics Sweden. The internal consistency of the RCADS‐25‐C scales was excellent for anxiety (mean 7.98 ± 5.93, omega 0.88) and depression (mean 6.37 ± 4.66, omega 0.87). Descriptive statistics, including a correlation table, are presented in Table 1.

**TABLE 1:** Descriptive statistics for RCADS‐25‐C anxiety and depression: Screen time, physical activity and sleep.

Measure	Mean	SD	Omega	Correlations^a^				
				1	2	3	4	5
1. Anxiety^b^	7.98	5.93	0.88					
2. Depression^b^	6.37	4.66	0.87	0.71				
3. Screen time	3.66	1.02	—	0.16	0.26			
4. Physical activity	4.19	1.71	—	−0.14	−0.20	−0.025		
5. Sleep	3.26	0.97	—	−0.17	−0.25	−0.35	0.08	

*Note:* Mean value of approximately 2–3 h of daily screen time on the ordinal scale. Mean value of approximately 90 min of physical activity on the ordinal scale. Mean value of approximately 10 h of sleep on the ordinal scale(s). SD, standard deviations.

Abbreviation: SD, standard deviations.

^a^ All correlations significant at *p* < 0.001.

^b^ Raw score.

**FIGURE 2:**
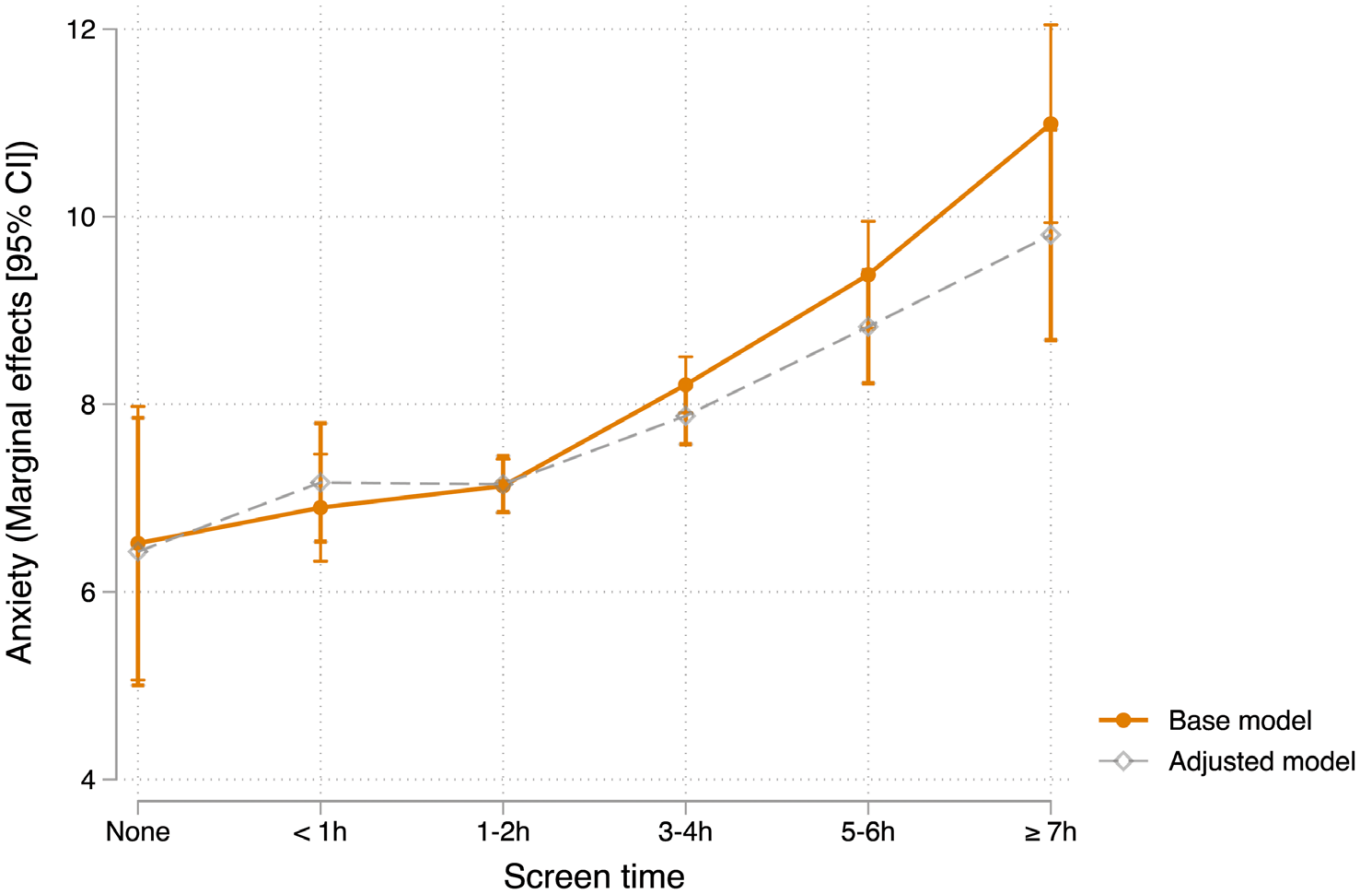
Plot of marginal effects of screen time on anxiety (base model) and plot of marginal effects of screen time on anxiety, adjusted for physical activity, sleep, sex, age, parents' education and household income (adjusted model).

An ordinary least squares regression analysis shows that screen time was significantly associated with anxiety (beta = 0.17, *p* < 0.001), but each ordinal group of screen time did not show significant associations with anxiety. The slope of marginal effects (Figure 2) was flattened when we included physical activity, sleep, sex, age, parents' education and household income. The results show that there appeared to be a threshold of around 1–2 h of screen time per day. After that, the slope got steeper, and each incremental ordinal step of the scale was significantly associated with a higher degree of anxiety symptoms. The full regression model revealed that greater levels of anxiety were significantly higher among girls and were also associated with less sleep and physical activity and lower household income (Table S1).

**FIGURE 3:**
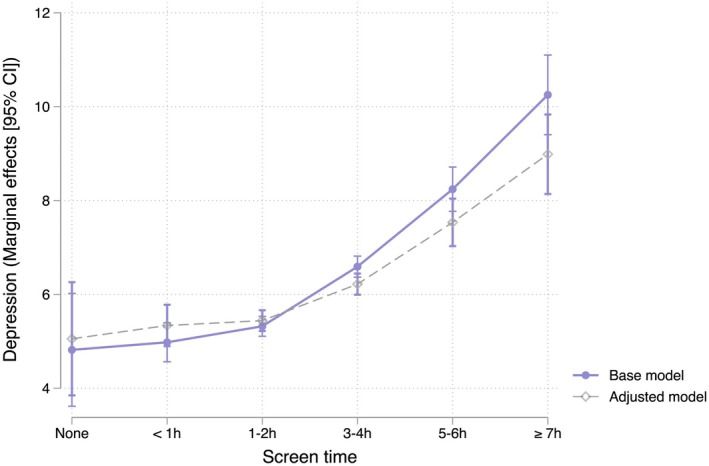
Plot of marginal effects of screen time on depression (base model) and plot of marginal effects of screen time on depression, when physical activity, sleep, sex, age, parents' education and household income were held constant (adjusted model).

A second ordinary least squares (OLS) regression analysis showed that daily screen time was significantly associated with depression (β = 0.27, *p* < 0.001). Each ordinal group of screen time was not significantly related to depression and this was in common with the findings on anxiety previously mentioned. We could see that the slope of marginal effects (Figure 3) was less steep when we included physical activity, sleep, sex, age, parents' education and household income. The same pattern emerged when we established a clear threshold in incrementally higher depression symptoms if screen time exceeded around 1–2 h per day. Furthermore, the covariates in the regression model demonstrated that higher levels of depression were significantly associated with girls, higher age, less sleep, less physical activity and lower household income (Table S2).

The scores for anxiety and depression nearly doubled when the daily screen time increased from approximately two hours to seven hours or more. However, statistically significant marginal effects were only observed when screen time exceeded two hours per day (Table S2).

## 4 Discussion

The main aim of this paper was to explore how screen time was associated with mental health among children aged 8–17 years in Sweden. We saw increased symptoms of anxiety and depression once the children had passed 1–2 h of screen time per day and this highlighted a possible existence danger zone of digital engagement. The finding partially confirmed the Goldilocks hypothesis, which suggests that it is important to find the right level that is beneficial for children. In other words, not too high and not too low. We also found evidence for the displacement hypothesis. This suggests that the negative associations between screen time and mental health that we observed may have displaced more beneficial activities, like physical activity and sleep. The associations were only marginally reduced in our models, which may indicate that less physical activity and sleep only explained a small part of the observed relationship.

Our general finding that screen time was negatively associated with mental health agreed with most studies [3, 4, 5, 6, 7, 9, 10, 24]. In particular, we observed similar incremental patterns to those reported in studies by Przybylski and Weinstein [2], Twenge and Campbell [25] and Rosenthal et al. [26]. Those studies also found that mental health symptoms rose after approximately two hours of screen time per day. In addition, we found the same curvilinear relationship that suggested that mental health symptoms may have increased exponentially for each additional hour of daily screen time. In contrast to three papers [2, 25, 26], we did not observe a J‐shaped function that reflected that mental health was elevated if there was no screen time at all.

Two important covariates were included in our models to test the displacement hypothesis. Our findings indicated an association between mental health issues and less sleep, in common with other studies [14, 15, 16]. We also found associations between higher levels of anxiety and depression and lower physical activity levels, echoing the conclusions of other studies [12, 13]. The adverse impact of screen time remained, even after adjusting for sleep and physical activity. This robustness persisted when controlling for sex, age, parents' education and household income. However, our attempt to challenge the displacement hypothesis should be approached with caution, because our analysis relied on self‐reported data. The displacement effect may have been more pronounced if we had used objective physical measurements and compositional analyses.

The increase in anxiety and depression scores when screen time exceeded 1–2 h may indicate that these children experienced more frequent or severe symptoms. However, we were not able to ascertain whether seven hours or more of screen time was associated with clinically relevant mental health deterioration. Despite this, we argue that those who engaged in excessive screen time daily may have been more vulnerable to developing mental health problems. Another point worth highlighting is that associations between increased mental health symptoms and higher levels of screen time usage may differ in other study populations. One example could be patients who have already been diagnosed with anxiety and depression.

Formulating precise guidelines on optimal screen time for children is challenging. Our empirical evidence suggests that the threshold for excessive screen time may be approximately two hours per day, as reported symptoms of anxiety and depression increased after that point. This may indicate that children are entering a danger zone for mental health problems. Screen time content may also be an essential element in the danger that children face. For example, scrolling through social media for two hours a day may be more strongly associated with poorer mental health than two hours playing video games. These findings highlight the need for a nuanced approach to recommendations and guidelines. For now, we suggest that parents try to be more involved in their children's screen time, by monitoring content rather than minutes and emphasising different types of activities. These could include interactive, creative and socially enriching types of screen engagement. After all, screen time can be an opportunity for family time, rather than a source of conflict or isolation. Likewise, screen time should probably not be used as a reward or punishment. Screens are here to stay and we need strategies that are built on cooperation, respect and understanding about how each generation makes the best of these screens.

### 4.1 Strengths and Limitations

A strength of this study was the use of a large, probability‐based sample of Swedish children aged 8–17, with a sample distribution that closely matched the target population for sex, age and geographical region [23]. Another strength was that the symptoms of anxiety and depression were measured with a validated instrument, namely the RCADS‐25‐C, which has shown strong psychometric properties and clinical relevance [20, 21, 22]. The systematic reviews and meta‐analyses only identified one published study that investigated the relationship between screen time and mental health with RCADS instruments. This reported positive longitudinal associations between different types of screen time and emotional disorders [27]. That makes our study even more relevant.

Our study had a number of limitations. One was the use of self‐reported measures, as only the self‐reported physical activity question had been validated through an extension study using accelerometer data [23]. This was an important limitation because self‐reports are often prone to several types of errors and biases and to issues regarding the quality of the instruments and survey questions. However, it is harder to ascertain how our findings may have been affected by psychological biases. For example, social desirability bias may have caused respondents to answer in line with certain socially accepted norms. The risk of recall bias could have been mitigated by specifically asking about screen time and physical activity the previous day. Having said that, this methodological choice also risks seemingly random events, such as binge‐watching during sick leave from school.

One of the inherent limitations in correlational studies like ours is that we could not show causality. Our theoretical arguments were based on previous research and we hypothesised that excessive screen time could have led to a higher degree of mental health symptoms. However, reversed causality was also possible, as a higher degree of mental health symptoms may have made subjects engage in more screen time. This potentially bi‐directional association was further clouded by acknowledging how different types of screen time may have had distinct effects. Studies that have distinguished between passive and active forms of screen time have suggested differential associations with mental health outcomes [28]. For example, scrolling through social media has been linked to poorer mental health [29]. In contrast, certain video games may be beneficial and enhance cognitive function [30, 31]. In addition, it has been suggested that screen time quality could be a better way of looking at the harmful effects of screen time [4, 28, 31]. We only present a crude measure of estimated screen time in this study but the 2022 Generation Pep Study presented more precise data [23]. The children were asked how they had spent their time online and multiple responses were possible [23]. The most popular activity was watching clips and reels, which was reported by 64% of the cohort, followed by gaming (42%) and social media (37%). Screen time quality may be more important than just screen time, but there has been little evidence to suggest that general screen time quality was high. Finally, although we used random probability sampling, our cohort was highly representative of the Swedish population in terms of sex, age and place of residence. However, families with lower educational attainment and household income were less likely to respond to the study [23]. This which may have limited the generalisability of our findings at the lower end of the socioeconomic scale.

## 5 Conclusion

This population‐based study of Swedish children aged 8**–**17 years found that screen time may be linked to poorer mental health when children exceed about two hours per day. Our findings showed higher levels of symptoms of anxiety and depression above this level. These associations remained, even after we accounted for the plausible displacement effects of reduced physical activity and sleep.

## Author Contributions

**Anders Carlander:** investigation, writing – original draft, methodology, validation, visualization, writing – review and editing, software, formal analysis, resources, conceptualization. **Sophie Cassel:** conceptualization, investigation, data curation, formal analysis, methodology, writing – review and editing. **Malin J‐son Höök:** conceptualization, investigation, writing – review and editing. **Oskar Lundgren:** supervision, writing – review and editing. **Ann‐Sophie Lindqvist Bagge:** writing – review and editing. **Marie Löf:** conceptualization, funding acquisition, project administration, supervision, writing – review and editing. [Correction added on 20 April 2026, after first online publication: The CRediT roles for the author ‘Oskar Lundgren’ have been included in this version.]

## Funding

The data collection was funded by the Swedish Heart Lung Foundation.

## Conflicts of Interest

The authors declare no conflicts of interest.

## Supporting information


**Table S1:** Regression of screen time on anxiety (Model 1), of screen time on anxiety when sex, age, parents' education, household income, physical activity and sleep were held constant (Model 2). Marginal effects from each model are included.
**Table S2:** Regression of screen time on depression (Model 1), of screen time on depression when sex, age, parents' education, household income, physical activity and sleep were held constant (Model 2). Marginal effects from each model are included.

## Data Availability

The data that support the findings of this study are openly available in Pep‐rapporten at https://researchdata.se/sv/catalogue/collection/the‐pep‐study.
