# Supplementary material for: Survey Showed That Limiting Daily Screen Time Could Help to Avoid Mental Health Issues in Children Aged 8–17Years
Source: Acta Paediatr. 2026 Mar 31;115(7):1538–44. doi: 10.1111/apa.70524 (PMC13250957; doi:10.1111/apa.70524)
Supplement: Supplementary file 1 — Table S1: Regression of screen time on anxiety (Model 1), of screen time on anxiety when sex, age, parents' education, household income, physical activity and sleep were held constant (Model 2). Marginal effects from each model are included. Table S2: Regression of screen time on depression (Model 1), of screen time on depression when sex, age, parents' education, household income, physical activity and sleep were held constant (Model 2). Marginal effects from each model are included. [file APA-115-1538-s001.docx]

**Supplementary material | Acta Paediatrica**

**Table S1. Regression of screen time on anxiety (Model 1), of screen time on anxiety when sex, age, parents’ education, household income, physical activity, and sleep were held constant (Model 2). Marginal effects from each model are included.**

|  | Model 1 | | | | | | Model 2 | | | | | |
| --- | --- | --- | --- | --- | --- | --- | --- | --- | --- | --- | --- | --- |
| Screen | B | SE^a^ | β | p | ME^b^ | SE^c^ | B | SE | β | p | ME^b^ | SE^c^ |
| None |  |  |  |  | 6.52 | .74 |  |  |  |  | 6.43 | .73 |
| <1h | .38 | .80 | .02 | .634 | 6.52 | .74 | .74 | .78 | .04 | .348 | 7.17 | .32 |
| 1-2h | .61 | .76 | .05 | .420 | 7.13 | .15 | .72 | .73 | .06 | .330 | 7.15 | .15 |
| 3-4h | 1.69 | .76 | .14 | .02 | 8.21 | .15 | 1.45 | .74 | .12 | .052 | 7.88 | .15 |
| 5-6h | 2.86 | .80 | .16 | <.001 | 9.38 | .29 | 2.40 | .80 | .13 | .003 | 8.83 | .31 |
| ≥7h | 4.47 | .92 | .16 | <.001 | 11 | .53 | 3.38 | .93 | .12 | <.001 | 9.81 | .57 |
|  |  |  |  |  |  |  |  |  |  |  |  |  |
| Girls |  |  |  |  |  |  | 3.04 | .19 | .27 | <.001 |  |  |
| Age |  |  |  |  |  |  | .03 | 0.4 | .01 | .555 |  |  |
| Education |  |  |  |  |  |  | .14 | .09 | .03 | .106 |  |  |
| Income |  |  |  |  |  |  | -.14 | .05 | -.06 | .003 |  |  |
| PA^d^ |  |  |  |  |  |  | -.19 | .06 | -.06 | .002 |  |  |
| Sleep |  |  |  |  |  |  | -.59 | .14 | -.1 | <.001 |  |  |
|  | F(5, 3920) = 21.09, p < .001, R² = .03 | | | | | | F(11, 3283) = 33.65, p < .001, R² = .12 | | | | | |

^a^Robust standard error (HC3)

^b^Marginal effect

^c^Delta-method standard error

^b^Physical activity

**Table S2. Regression of screen time on depression (Model 1), of screen time on depression when sex, age, parents’ education, household income, physical activity, and sleep were held constant (Model 2). Marginal effects from each model are included.**

|  | Model 1 | | | | | | Model 2 | | | | | |
| --- | --- | --- | --- | --- | --- | --- | --- | --- | --- | --- | --- | --- |
| Screen | B | SE^a^ | β | p | ME^b^ | SE^c^ | B | SE | β | p | ME^b^ | SE^c^ |
| None |  |  |  |  | 4.82 | .61 |  |  |  |  | 5.05 | .62 |
| <1h | .16 | .65 | .01 | .804 | 4.98 | .21 | .28 | .64 | .02 | .660 | 5.34 | .23 |
| 1-2h | .50 | .62 | .05 | .419 | 5.32 | .11 | .39 | .62 | .04 | .533 | 5.44 | .11 |
| 3-4h | 1.78 | .62 | .19 | .004 | 6.59 | .11 | 1.16 | .63 | .13 | .063 | 6.22 | .11 |
| 5-6h | 3.42 | .66 | .24 | <.001 | 8.24 | .24 | 2.48 | .68 | .18 | <.001 | 7.53 | .26 |
| ≥7h | 5.43 | .75 | .25 | <.001 | 10.25 | .43 | 3.93 | .76 | .18 | <.001 | 8.99 | .43 |
|  |  |  |  |  |  |  |  |  |  |  |  |  |
| Girls |  |  |  |  |  |  | 1.79 | .14 | .20 | <.001 |  |  |
| Age |  |  |  |  |  |  | .10 | .03 | .06 | .005 |  |  |
| Education |  |  |  |  |  |  | -.03 | .07 | -.01 | .691 |  |  |
| Income |  |  |  |  |  |  | -.09 | .04 | -.05 | .014 |  |  |
| PA^d^ |  |  |  |  |  |  | -.22 | .05 | -.08 | <.001 |  |  |
| Sleep |  |  |  |  |  |  | -.62 | .11 | -.13 | <.001 |  |  |
|  | F(5, 3931) = 54.70, p < .001, R² = .08 | | | | | | F(11, 3292) = 46.38, p < .001, R² = .17 | | | | | |

^a^Robust standard error (HC3)

^b^Marginal effect

^c^Delta-method standard error

^b^Physical activity
